# Supplementary material for: Characterization of Putative Sporulation and Germination Genes in Clostridium perfringens Food-Poisoning Strain SM101
Source: Microorganisms. 2022 Jul 22;10(8):1481. doi: 10.3390/microorganisms10081481 (PMC9332280; doi:10.3390/microorganisms10081481)
Supplement: Supplementary file 1 [file microorganisms-10-01481-s001.zip › microorganisms-1810147-supplementary.pdf]

Supplementary Data

Supplementary Table S1. *C. perfringens* strains used in this study.

Strains	Relevant characteristic(s)	Reference
SM101	Wild-type strain; a transformable derivative of NCTC8798, a food-poisoning type F *C. perfringens* human strain; carries a chromosomal *cpe*	[1]
IH101	Δ*spo0A* (*spo0A*::*catP*) mutant of SM101	[2,3]
NM101	Δ*sigE* (*sigE*::*catP*) mutant of SM101	[4]
PKT136	Δ*ylmC* (*ylmC*::*ermB*) mutant of SM101	This study
PKT137	Δ*bkdR* (*bkdR*::*ermB*) mutant of SM101	This study
PKT139	Δ*yyaC1* (*yyaC1*::*ermB*) mutant of SM101	This study
PKT140	Δ*yyaC2* (*yyaC2*::*ermB*) mutant of SM101	This study
PKT141	Δ*ytaF* (*ytaF*::*ermB*) mutant of SM101	This study
PKT142	Δ*ytxC* (*ytxC*::*ermB*) mutant of SM101	This study
SM101(pPKT2)	Wild-type strain carrying P_{ylmC}-*gusA* fusion	This study
SM101(pPKT6)	Wild-type strain carrying P_{bkdR}-*gusA* fusion	This study
SM101(pPKT10)	Wild-type strain carrying P_{ytaF}-*gusA* fusion	This study
SM101(pPKT12)	Wild-type strain carrying P_{ylxA}-*gusA* fusion	This study
SM101(pPKT14)	Wild-type strain carrying P_{ylzA}-*gusA* fusion	This study
SM101(pPKT16)	Wild-type strain carrying P_{ytxC}-*gusA* fusion	This study
SM101(pPKT18)	Wild-type strain carrying P_{yyaC1}-*gusA* fusion	This study
SM101(pPKT20)	Wild-type strain carrying P_{yyaC2}-*gusA* fusion	This study
PKT140 (pPKT130)	Δ*yyaC2* mutant complemented with wild-type *yyaC2*	This study
PKT142 (pPKT125)	Δ*ytxC* mutant complemented with wild-type *ytxC*	This study

Supplementary Table S2. List of primers used in this study.

Primer name	Primer sequence (5'-3')[a]	Gene	Nucleotide position[b]	Use[c]
CPP1016	GCA GCG TCG ACT ATA TAT AAC AGC AAT TTG CGT GCC	*bkdR*	-383 to -358	GUS
CPP1017	GAC GCT GCA GAG TAT CTA GTG TGT GTA ATC CTA AGG	*bkdR*	+31 to +55	GUS
CPP1020	GCA GCG TCG ACG GAA CCT ACT GTA TCT CAA ATA GC	*ylmC*	-501 to -477	GUS
CPP1021	GAC GCT GCA GCC TTC ACT TAC ATC TAT CAC TTC C	*ylmC*	+41 to +65	GUS
CPP1018	GCA GCG TCG ACA AGA AGA ATA GTA CCT TAT CTC C	*ylxY*	-348 to -325	GUS
CPP1019	GAC GCT GCA GTG CCC AAT TCA CAT CAA AGG	*ylxY*	+166 to +186	GUS
CPP1022	GCA GCG TCG ACG AAG GTG AGA AAT TAG CTA GTG	*ylzA*	-441 to -419	GUS
CPP1023	GAC GCT GCA GTA AGC ATA CCT CTA TCT CTA GC	*ylzA*	+108 to +130	GUS
CPP1033	GCA GCG TCG ACG ATC ACA TAA GTA ATA GAG AAA GCC	*ytaF*	-231 to -206	GUS
CPP1034	GAC GCT GCA GGT TCC TAA AGC AGA TAT GGA TGC	*ytaF*	+117 to +140	GUS
CPP1162	GCA GCG TCG ACT CTG GAT GAC CAT CTA TAG CA	*ytxC*	-262 to -241	GUS
CPP1163	GAC GCT GCA GCT GAG ATA CTT TCT GAC ACA CCA A	*ytxC*	+94 to +117	GUS
CPP1182	GCA GCG TCG ACT TTT GAT GAT TCC TCC TCA GC	*yyaC1*	-468 to -447	GUS
CPP1183	GAC GCT GCA GGG TAC CAA CTA ATG GAC CTA AGC A	*yyaC1*	+126 to +150	GUS
CPP1184	GCA GCG TCG ACC CCT GTG CTA GGT ATG CCC TT	*yyaC2*	-332 to -311	GUS
CPP1185	GAC GCT GCA GAT CTC CTG TGG AGC GAT CAG A	*yyaC2*	+126 to +146	GUS
CPP1210	AAA AAA GCT TAT AAT TAT CCT TAC AGG TCC TTG AAG TGC GCC CAG ATA GGG TG	*bkdR*	IBS 357/358s	MP
CPP1211	CAG ATT GTA CAA ATG TGG TGA TAA CAG ATA AGT CCT TGA AAG TAA CTT ACC TTT CTT TGT	*bkdR*	EBS1d 357/358s	MP
CPP1212	TGA ACG CAA GTT TCT AAT TTC GGT TAC CTG TCG ATA GAG GAA AGT GTC T	*bkdR*	EBS2 357/358s	MP
CPP1207	AAA AAA GCT TAT AAT TAT CCT TAA GAA TCT TAT CTG TGC GCC CAG ATA GGG TG	*ylmC*	IBS 126/127s	MP
CPP1208	CAG ATT GTA CAA ATG TGG TGA TAA CAG ATA AGT CTT ATC TAT TAA CTT ACC TTT CTT TGT	*ylmC*	EBS1d 126/127s	MP
CPP1209	TGA ACG CAA GTT TCT AAT TTC GAT TAT TCT TCG ATA GAG GAA AGT GTC T	*ylmC*	EBS2 126/127s	MP
CPP1186	AAA AAA GCT TAT AAT TAT CCT TAA GGG TCC TTG AAG TGC GCC CAG ATA GGG TG	*ytaF*	IBS 98/99a	MP
CPP1187	CAG ATT GTA CAA ATG TGG TGA TAA CAG ATA AGT CCT TGA AAA TAA CTT ACC TTT CTT TGT	*ytaF*	EBS1d 98/99a	MP
CPP1188	TGA ACG CAA GTT TCT AAT TTC GAT TAC CCT TCG ATA GAG GAA AGT GTC T	*ytaF*	EBS2 98/99a	MP
CPP1189	AAA AAA GCT TAT AAT TAT CCT TAG TAG ACG ATT ATG TGC GCC CAG ATA GGG TG	*ytxC*	IBS 519/520s	MP
CPP1190	CAG ATT GTA CAA ATG TGG TGA TAA CAG ATA AGT CGA TTA TAT TAA CTT ACC TTT CTT TGT	*ytxC*	EBS1d 519/520s	MP
CPP1191	TGA ACG CAA GTT TCT AAT TTC GAT TTC TAC TCG ATA GAG GAA AGT GTC T	*ytxC*	EBS2 519/520s	MP
CPP1216	AAA AAA GCT TAT AAT TAT CCT TAA CCA TCA TAG TAG TGC GCC CAG ATA GGG TG	*yyaC1*	IBS 96/97s	MP
CPP1217	CAG ATT GTA CAA ATG TGG TGA TAA CAG ATA AGT CAT AGT ATG TAA CTT ACC TTT CTT TGT	*yyaC1*	EBS1d 96/97s	MP
CPP1218	TGA ACG CAA GTT TCT AAT TTC GAT TAT GGT TCG ATA GAG GAA AGT GTC T	*yyaC1*	EBS2 96/97s	MP
CPP1219	AAA AAA GCT TAT AAT TAT CCT TAG TAG GCC ACG TAG TGC GCC CAG ATA GGG TG	*yyaC2*	IBS 342/343s	MP
CPP1220	CAG ATT GTA CAA ATG TGG TGA TAA CAG ATA AGT CCA CGT ACT TAA CTT ACC TTT CTT TGT	*yyaC2*	EBS1d 342/343s	MP

CPP1221	TGA ACG CAA GTT TCT AAT TTC GAT TCC TAC TCG ATA GAG GAA AGT GTC T	*yyaC2*	EBS2 342/343s	MP
	CGA AAT TAG AAA CTT GCG TTC AGT AAA C		EBS Universal	MP
CPP1031	GCT TGC AAT ACT TTC TCT TAA GGT GT	*bkdR*		Detect
CPP1204	TGA GGA AGC TGA GAT GAT GGA	*bkdR*		Detect
CPP1092	GCG GGT ACC CGG TAT ATC TCA AGC GCA AGT TTC	*ylmC*		Detect
CPP1094	GCA GCG TCG ACA TCC ACG CCT TTC TCT AAG TTG	*ylmC*		Detect
CPP1194	TGG AGG CAG AAA AAG TGA GC	*ytaF*		Detect
CPP1195	GGG CTA AGG ATA AGG CTA GGG	*ytaF*		Detect
CPP1196	TCT CAT ATG GAA CTC ACT TCG TT	*ytxC*		Detect
CPP1197	TCC TAC TTC AGC AGC ATC TGT	*ytxC*		Detect
CPP1182	GCA GCG TCG ACT TTT GAT GAT TCC TCC TCA GC	*yyaC1*		Detect
CPP1183	GAC GCT GCA GGG TAC CAA CTA ATG GAC CTA AGC A	*yyaC1*		Detect
CPP1202	TCT GAT CGC TCC ACA GGA GA	*yyaC2*		Detect
CPP1203	GCT TAA GTC TCC TAC TTC AGG CA	*yyaC2*		Detect
CPP1198	TCT GGA TGA CCA TCT ATA GCA	*ytxC*		CP
CPP1199	GCA GCG TCG ACG CTG TAA GGT GAG AAA CGG C	*ytxC*		CP
CPP1236	GCG GGT ACC CCC CTG TGC TAG GTA TGC CCT T	*yyaC2*		CP
CPP1237	GCA GCG TCG ACA GCT TTC GAA GAC GTT GGA AGT	*yyaC2*		CP

[a]Restriction sites are underlined.

[b]Nucleotide numbering begins at the first base of the translation codon of the relevant gene.

[c] GUS, construction of plasmid for GUS assay; MP, construction of mutator plasmid; Detect, primers used for the detection of mutant isolates; CP, construction of complemented plasmid

Supplementary Table S3. List of plasmids used in this study.

Plasmids	Relevant characteristic(s)	Reference
pJIR3566	*C. perfringens* targetron plasmid derived from pJIR750ai, contains *ermB*::RAM and lacZα; Cm^R	[5]
pMRS127	*C. perfringens*/*E. coli* shuttle vector carrying a promoterless *gusA*; Em^R	[6]
pJIR750	*C. perfringens*/*E. coli* shuttle vector; Cm^R	[7]
pPKT1	a 459–bp of putative promoter of *ylmC* cloned into pCR-XL-TOPO; Kan^R	This study
pPKT2	a 459–bp of putative promoter of *ylmC* from pPKT1 cloned into pMRS127; Em^R	This study
pPKT5	a 389–bp of putative promoter of *bkdR* cloned into pCR-XL-TOPO; Kan^R	This study
pPKT6	a 389–bp of putative promoter of *bkdR* from pPKT5 cloned into pMRS127; Em^R	This study
pPKT9	a 435–bp of putative promoter of *ytaF* cloned into pCR-XL-TOPO; Kan^R	This study
pPKT10	a 435–bp of putative promoter of *ytaF* from pPKT3 cloned into pMRS127; Em^R	This study
pPKT11	a 321–bp of putative promoter of *ylxY* cloned into pCR-XL-TOPO; Kan^R	This study
pPKT12	a 321–bp of putative promoter of *ylxY* from pPKT3 cloned into pMRS127; Em^R	This study
pPKT13	a 523–bp of putative promoter of *ylzA* cloned into pCR-XL-TOPO; Kan^R	This study
pPKT14	a 523–bp of putative promoter of *ylzA* from pPKT3 cloned into pMRS127; Em^R	This study
pPKT15	a 478–bp of putative promoter of *ytxC* cloned into pCR-XL-TOPO; Kan^R	This study
pPKT16	a 478–bp of putative promoter of *ytxC* from pPKT3 cloned into pMRS127; Em^R	This study
pPKT17	a 456–bp of putative promoter of *yyaC1* cloned into pCR-XL-TOPO; Kan^R	This study
pPKT18	a 456–bp of putative promoter of *yyaC1* from pPKT3 cloned into pMRS127; Em^R	This study
pPKT19	a 321–bp of putative promoter of *yyaC2* cloned into pCR-XL-TOPO; Kan^R	This study
pPKT20	a 321–bp of putative promoter of *yyaC2* from pPKT3 cloned into pMRS127; Em^R	This study
pPKT121	a 350–bp group-II intron retargeted to *ytaF* cloned into pCR-XL-TOPO; Kan^R	This study
pPKT122	a 350–bp group-II intron retargeted to *ytxC* cloned into pCR-XL-TOPO; Kan^R	This study
pPKT131	a 350–bp group-II intron retargeted to *ylmC* cloned into pCR-XL-TOPO; Kan^R	This study
pPKT132	a 350–bp group-II intron retargeted to *bkdR* cloned into pCR-XL-TOPO; Kan^R	This study
pPKT134	a 350–bp group-II intron retargeted to *yyaC1* cloned into pCR-XL-TOPO; Kan^R	This study
pPKT135	a 350–bp group-II intron retargeted to *yyaC2* cloned into pCR-XL-TOPO; Kan^R	This study
pPKT123	*ytaF* intron product from pPKT121 cloned into pJIR3566 (*ytaF* mutator plasmid); Cm^R	This study
pPKT124	*ytxC* intron product from pPKT122 cloned into pJIR3566 (*ytxC* mutator plasmid); Cm^R	This study
pPKT136	*ylmC* intron product from pPKT131 cloned into pJIR3566 (*ylmC* mutator plasmid); Cm^R	This study
pPKT137	*bkdR* intron product from pPKT132 cloned into pJIR3566 (*bkdR* mutator plasmid); Cm^R	This study
pPKT139	*yyaC1* intron product from pPKT134 cloned into pJIR3566 (*yyaC1* mutator plasmid); Cm^R	This study
pPKT140	*yyaC2* intron product from pPKT135 cloned into pJIR3566 (*yyaC2* mutator plasmid); Cm^R	This study

References

1. Zhao, Y.; Melville, S.B. Identification and characterization of sporulation-dependent promoters upstream of the enterotoxin gene (*cpe*) of *Clostridium perfringens*. *J Bacteriol* **1998**, *180*, 136-142.
2. Huang, I.H.; Waters, M.; Grau, R.R.; Sarker, M.R. Disruption of the gene (*spo0A*) encoding sporulation transcription factor blocks endospore formation and enterotoxin production in enterotoxigenic *Clostridium perfringens* type A. *FEMS Microbiol Lett* **2004**, *233*, 233-240, doi:10.1016/j.femsle.2004.02.014.
3. Huang, I.H.; Sarker, M.R. Complementation of a *Clostridium perfringens* spo0A mutant with wild-type spo0A from other *Clostridium* species. *Appl Environ Microbiol* **2006**, *72*, 6388-6393, doi:10.1128/AEM.02218-05.
4. Paredes-Sabja, D.; Sarker, N.; Sarker, M.R. *Clostridium perfringens tpeL* is expressed during sporulation. *Microb Pathog* **2011**, *51*, 384-388, doi:10.1016/j.micpath.2011.05.006.
5. Cheung, J.K.; Keyburn, A.L.; Carter, G.P.; Lanckriet, A.L.; Van Immerseel, F.; Moore, R.J.; Rood, J.I. The VirSR two-component signal transduction system regulates NetB toxin production in *Clostridium perfringens*. *Infect Immun* **2010**, *78*, 3064-3072, doi:10.1128/IAI.00123-10.
6. Raju, D.; Waters, M.; Setlow, P.; Sarker, M.R. Investigating the role of small, acid-soluble spore proteins (SASPs) in the resistance of *Clostridium perfringens* spores to heat. *BMC Microbiol* **2006**, *6*, 50, doi:10.1186/1471-2180-6-50.
7. Bannam, T.L.; Rood, J.I. *Clostridium perfringens-Escherichia coli* shuttle vectors that carry single antibiotic resistance determinants. *Plasmid* **1993**, *29*, 233-235, doi:10.1006/plas.1993.1025.
